# Intestinal Organoids: New Tools to Comprehend the Virulence of Bacterial Foodborne Pathogens

**DOI:** 10.3390/foods11010108

**Published:** 2022-01-01

**Authors:** Mayra Aguirre Garcia, Killian Hillion, Jean-Michel Cappelier, Michel Neunlist, Maxime M. Mahe, Nabila Haddad

**Affiliations:** 1 UMR SECALIM, INRAE, Oniris, 44307 Nantes, France; maaguirreg@unal.edu.co (M.A.G.); jean-michel.cappelier@oniris-nantes.fr (J.-M.C.); 2 UMR Inserm 1235-TENS, INSERM, Université de Nantes, Institut des Maladies de l’Appareil Digestif–CHU de Nantes, 44035 Nantes, France; killian.hillion@univ-nantes.fr (K.H.); michel.neunlist@univ-nantes.fr (M.N.); maxime.mahe@cchmc.org (M.M.M.); 3 Department of Pediatric General and Thoracic Surgery, Cincinnati Children’s Hospital Medical Center, Cincinnati, OH 45229, USA; 4 Department of Pediatrics, University of Cincinnati, Cincinnati, OH 45220, USA

**Keywords:** pathogenic mechanism, foodborne bacteria, in vitro cell models, organoids, enteroids

## Abstract

Foodborne diseases cause high morbidity and mortality worldwide. Understanding the relationships between bacteria and epithelial cells throughout the infection process is essential to setting up preventive and therapeutic solutions. The extensive study of their pathophysiology has mostly been performed on transformed cell cultures that do not fully mirror the complex cell populations, the in vivo architectures, and the genetic profiles of native tissues. Following advances in primary cell culture techniques, organoids have been developed. Such technological breakthroughs have opened a new path in the study of microbial infectious diseases, and thus opened onto new strategies to control foodborne hazards. This review sheds new light on cellular messages from the host–foodborne pathogen crosstalk during in vitro organoid infection by the foodborne pathogenic bacteria with the highest health burden. Finally, future perspectives and current challenges are discussed to provide a better understanding of the potential applications of organoids in the investigation of foodborne infectious diseases.

## 1. Introduction

Foodborne diseases (FBDs) are thought to be a major public health issue that contributes significantly to human morbidity and mortality around the world. The World Health Organization (WHO) estimates that almost one person in 10 falls ill from eating unsafe food every year [1]. Although the European region has the lowest burden in the world, the WHO calculated that more than 23 million people become sick annually because of FBDs [2]. Moreover, foodborne hazards of microbial origin raise a broad number of issues due to their economic burden. The European Food Safety Authority (EFSA) has estimated that the overall economic impact of human salmonellosis in Europe could be as high as EUR 3 billion annually [3]. In addition, antibiotic resistance and increasing food contamination as a consequence of environmental changes and dynamic methods of food production threaten to compound this problem further [4].

The surveillance of FBDs and our ability to tackle the knowledge gaps regarding host–pathogen–environment interactions need to be improved for the better prevention and control of microbial foodborne poisoning. Despite significant results from a large number of studies, their pathophysiology still appears to be poorly characterized, even less so where the pathogen can spread to distant organs and tissues through the blood stream and cause severe complications. One permanent challenge in this area of study is the lack of experimental models to address infection mechanisms and establish a clear picture of FBD biology.

To date, two-dimensional (2D) cultured cell lines have mostly been used, but the reproducibility of the overall physiology remains questionable. Organoids help to overcome the shortcomings of cell line monolayers thanks to their high cell type diversity and closer morphology to native intestinal tissue. They can be used to study the same questions as those addressed with monotypic cell systems, and many more. Organoids may be envisioned as a new tool that holds great promise for addressing novel challenges in the study of foodborne pathogens (FBPs)–host interactions. In this review, we describe the main advances in the field of FBPs relating to the use of organoid model systems and discuss their use for modeling bacterial FBDs, focusing on the foodborne bacteria with the highest disease burden.

## 2. Moving from Cell Lines to Intestinal Organoids

The oral route is the main entry site of FBPs, and the primary site of infection is the gastrointestinal tract [5]. They generally induce mild to severe enteritis, with widely known symptoms [6]. Because of this common pattern of infection, studies have been mostly focused on what occurs at the intestinal interface. The biology of these diseases remains less explored in other tissues [7], even though FBPs may occasionally spread deeply in the tissues and cause severe complications, permanent disability, and death [8,9,10]. 

From a historical perspective of model development and attempts to characterize bacterial FBP pathogenesis, concerns have emerged regarding animal models because bacterial intestinal pathogenesis varies considerably between humans and animals and the occurrence of symptoms in animals remains rare [11]. For example, *Campylobacter jejuni* and *Salmonella enterica*, both considered the main causes of bacterial FBDs worldwide, are mainly responsible for asymptomatic intestinal carriage in livestock [12]. In addition, national and international legislation and regulations restrict the use of animals in scientific procedures. The 3Rs principle (replacement, reduction, and refinement) aims to reduce the number of animals used in experimentation, which has led to the development of alternative methods [13]. In view of this, cell culture models of bacterial interaction with the epithelium have proved valuable for defining bacterium–host interactions [11]. 

**Figure 1:**
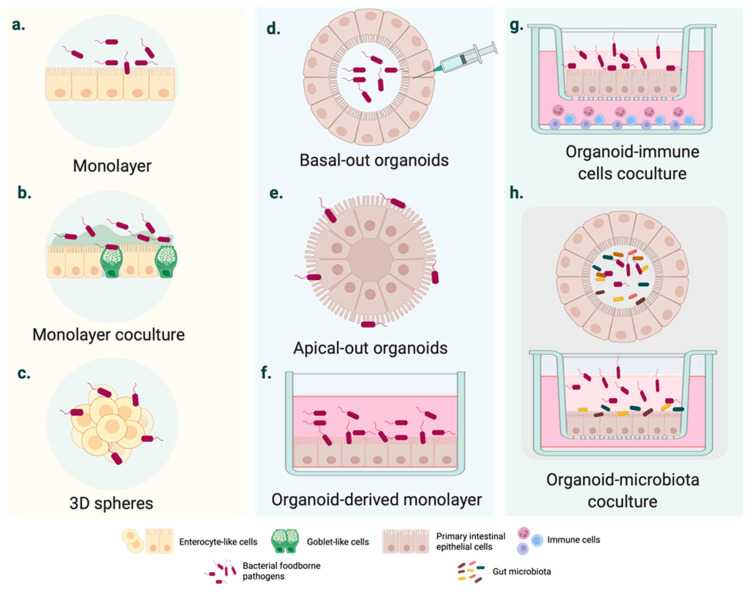
Cell culture systems mimicking intestinal FBD. (**a**–**c**) Intestinal FBD models derived from immortalized cells. (**a**) Polarized homogeneous cell monolayer typically based on immortalized cell lines with an enterocyte-like phenotype (e.g., Caco-2 cell monolayer). (**b**) Heterogeneous cell monolayer coculturing different cell lines to mimic essential intestinal features, such as the mucus- carrying intestinal tissue (e.g., Caco-2 and HT29 co-culture in vitro cell models). (**c**) 3D cell spheres developed from tumor-derived cell lines. (**d**–**f**) Intestinal organoid cultures generated from pluripotent stem cells (PSCs) or adult stem cells (AdSCs). (**d**) Basal-out organoid. The pathogen is generally injected inside the organoid. (**e**) Apical-out organoids might enhance the access of FBP with a high preference for the apical intestinal compartment. (**f**) Organoid-derived monolayers are D cell infection systems, such as the conventional immortalized cell cultures. (**g**–**h**) Coculture of intestinal organoids with immune cells and microbiota. More sophisticated organoid-based cultures, including intestinal epithelium–immune system and epithelium–microbiota interactions during infection.

The gold standard in intestinal modelling is based on immortalized cancer-derived cell lines, such as the enterocyte-like Caco-2 cell line. Numerous conclusions have been drawn from infected polarized or unpolarized cell monolayers (Figure 1a), even though it has been widely demonstrated over the last 50 years that these cell systems are outperformed [14]. As they consist of tumor-derived cells, they may not represent the native and healthy human intestine [15]. Several factors are likely to define intestinal homeostasis, and these vary considerably between cancer cell lines and the epithelial cells of native organs [16]. Structurally speaking, cell monolayers do not account for three-dimensional (3D) architecture and the complex cell population of the intestinal epithelium. 

In light of these disadvantages, cell coculture systems have been used to mirror the physiology of the human intestine more consistently. For instance, triple or cell coculture models (Figure 1b) have represented mucus-carrying intestinal tissue and basic elements of the innate immune system [17,18,19,20,21]. In parallel, the rotating wall vessel (RWV) facilitated the intestinal cell aggregation and growth in three dimensions (Figure 1c). Three-dimensional spheres resemble the native intestinal epithelium more accurately than monolayers derived from the same cell line [22]. The responses to bacterial pathogens also differ from those observed in 2D cell models [22,23].

**Figure 2:**
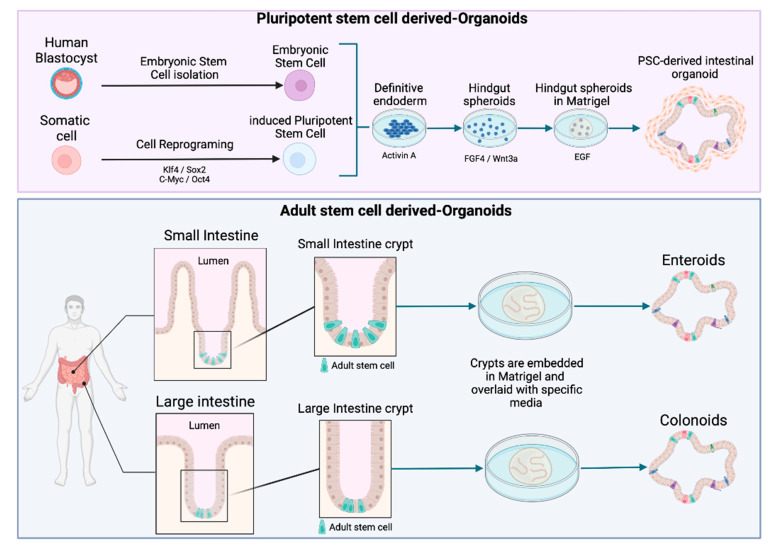
Schematic diagram of intestinal organoid, enteroid, and colonoid generation. Organoids can be derived from pluripotent stem cells (PSCs), including either induced pluripotent stem cells (iPSC) or embryonic stem cells (ESC). Enteroids and colonoids can be grown from the adult stem cells (AdSC) isolated from intestinal crypts.

Owing to the potential of organoids, the number of citations including the term “organoid” has rocketed in the last years. However, there does not seem to be a consensus on a general definition of organoids in the literature. In order to avoid misunderstandings, the recent definition suggested by Fujii and Sato was adopted in this review [24], i.e., ‘‘any heterotypic structures that can be reproducibly generated from single cells or cell clusters derived from somatic tissues or pluripotent stem cells, can self-assemble through cell–cell and cell–extracellular matrix (ECM) communications, and have some features of counterpart in vivo tissues’’ [24]. A further distinction is made according to the type of stem cell used to generate the organoids. While intestinal human organoids can be derived from pluripotent stem cells (PSCs) (including embryonic stem cells (ESCs) and induced pluripotent stem cells (iPSCs)) (Figure 2), adult stem cell (AdSC)-based organoids are initiated from self-renewing tissues, such as the gastrointestinal epithelium (see Figure 2) [25,26]. Two additional terms, enteroids and colonoids, are often used in the context of organoids to refer to the 3D models derived from intestinal and colon adult stem cells that only comprise epithelial cells (Figure 2) [27].

Contrary to immortalized cancer-derived cell lines, intestinal organoids are characterized by the capacity to generate crypt-like domains with proliferative regions able to differentiate into all of the epithelial cell lineages. They also possess villus-like domains able to maintain cellular polarization toward the tissue. A comparison of 2D versus 3D cell culture systems is provided in Table 1.

To mimic the architectural and physiological properties of the in vivo small intestine, the models for foodborne diseases require differentiated crypt-villus structures. Intestinal crypts contain stem cells, which maintain the epithelial progenitor cells pool. Once generated, epithelial cells migrate toward the lumen, and differentiate and die at the tip of the villi. This process leads to a complete regeneration of the intestinal epithelium every 4–5 days [31]. Organoid culture is based on the capacity of the intestinal epithelial stem cells to perpetually divide and produce epithelial progenitor cells. The discovery of *Lgr5* (Leucine-rich repeat-containing G protein-coupled receptor 5) has paved the way for culturing adult stem cells [32]. *Lgr5*^+^ intestinal stem cells cultured in 3D can undergo multi-lineage differentiation to ultimately form a “mini-gut”. In 2009, Sato et al. developed this long-term culture based on crucial signaling pathways, such as the Wnt/β-Catenin pathway and the EGF/EGF receptor (EGFR) with ECM-supported culture [33]. The resulting organoid culture system has been successfully applied to culture other epithelial organs, including stomach, pancreas, colon, and liver organoids [14].

Organoids have been mainly used for the study of cancer and genetic disorders as well as host cell–microorganism interactions [34]. In the organoid–pathogen coculture, several constraints in the mimicking of viral and human host-specific infections have been overcome. Alternatively, organoids generated from genetically modified pluripotent stem cells or from patients harboring mutations of clinical interest have opened a new window onto human infection diseases [35]. Furthermore, these practical and reproducible in vitro models of infection lead to the exploration of additional host–microbe dynamics, e.g., in disseminated infections [7,36,37].

Intestinal organoids usually form structures with budded and branched shapes [38], encapsulating the apical surface and the lumen (Figure 1d) [39]. This makes pathogen delivery inside the organoid interior more challenging from a technical point of view. Even though several studies have employed microinjection (Figure 1e), this is a tedious technique and observations can be disturbed by cellular material accumulating within the luminal side; moreover, cellular material may damage the organoid epithelium [39]. 

In 2019, Co et al. developed a culture system where organoids could precisely adopt polarity-specific parameters inspired by previous studies of polarity reversal in Madin–Darby canine kidney (MDCK) spheroids [39,40]. The resulting method provided a cell apparatus with an apical-out surface that promoted pathogen inclusion, especially of microbes with a marked preference for interacting with the apical intestinal compartment [39].

Although the study of intestinal epithelial cell (IEC)–pathogen interactions is time and cell consuming [39], most studies have used organoid-derived monolayers on insert/filter membranes (Figure 1f). Two-dimensional cell systems, as with other conventional transmembrane models, provide experimental access to the apical or the basolateral surface [41]. Similarly, monolayers of somatic cells allow adding other nearby intestinal cells to transformed cell lines in coculture to analyze the cellular crosstalk associated with the response to infection (Figure 1g) [42,43]. Although these complex cell systems are still in their infancy, advances have been made in modeling the intestinal microenvironment systems containing macrophages and T-cells (Figure 1g) [42,44] or microbiota (Figure 1h). On a wider scale, hybrid cell cultures could provide insights into the tissue inflammation and carcinogenesis significantly associated with intestinal infections. Table 2 summarizes the main advantages and disadvantages of 3D cell cultures.

## 3. Using Organoids to Explore the Cell and Tissue Tropism of FBPs

Regarding the infection capacity of FBPs, plausible discrepancies can be observed between homogenous cell monolayers and organoids that retain most of the intestinal cell composition and somatic signatures. Early works have shown that bacteria can cause the loss of a tissue’s structural integrity in intestinal organoids. Unsurprisingly, a growing body of evidence has assessed this common and fundamental issue. Antibiotic-protection assays coupled to confocal imaging to evaluate changes of the actin network have showed that *Salmonella*-, enterohemorrhagic *Escherichia coli* (EHEC)-, *Listeria monocytogenes*-, or *Shigella*-infected organoids showed intracellular pathogen carriage and damage of intestinal tissue in vitro [39,50,51,52]. 

Upon reaching the intestinal epithelium, some pathogens exhibit a higher affinity for regional intestinal segments [53]. Enteroids derived from cells from an anatomical region of the intestine could be a potential starting point for reliably studying segment-specific colonization on an in vitro device, an achievement never attained in whole animal models [54]. VanDussen et al. inoculated various strains of pathogenic *E. coli* to the apical surface of a cell monolayer generated from the dissociation of human intestinal biopsies [41]. *E. coli* EPEC strains preferentially adhered to ileal epithelial cells, whereas *E. coli* EAggEC and EHEC strains instead adhered to rectal epithelial cells. In et al. noted a remarkable difference between the number of EHEC bacteria associated with the apical surface in organoids representing colon and jejunum environments [51]. The authors indicated that the preference of EHEC for these colonoids could be related to the colon-specific differentiation [51]. Each *E. coli* pathotype usually possesses distinct virulence mechanisms to disrupt the host intestinal epithelium. Adherence patterns are one of the key signs generally accepted among *E. coli* pathovars [55]. Rajan et al. mimicked bacterial adhesion using enteroids made from crypts isolated from tissues from four different gut segments. Histopathological comparisons of infected enteroids suggested that *E. coli* EAggEC aggregated in several ways, including those patterns observed in classic in vitro models and new ones, with a remarkable dependency on donor and intestinal segment tropism [56].

Unlike EHEC, *Shigella flexneri* can invade enteroids from the duodenum, ileum, and colon in the same manner [57]. However, these findings substantially contrast with the in vivo shigellosis biology that describes a specificity of *Shigella* to the rectal and colonic mucosae [58]. Thus, other elements of the intestinal microenvironment, such as vasculature, the enteric nervous system, or the resident microbiota contributing human colon infection, were not taken into account with the previous enteroid study [57]. 

Several studies have showed the preferential attachment of FBP on the apical surface of immortalized cell lines [11,20,59,60,61]. However, some works have investigated the ability of enterocytes to internalize bacteria for transcellular translocation from the basolateral to the apical compartment. To address this issue, Co et al. developed a reversed polarity apical-out human enteroid model [39]. Thanks to this novel cell culture platform, they were able to compare the binding patterns of *S. enterica* Typhimurium and *L. monocytogenes*. *Salmonella* predominantly invade apical-out enteroids and induce cytoskeletal rearrangement, as described using cancer derived monolayers [62]. Conversely, the Gram-positive *L. monocytogenes* adhered more to the basal-out enteroids. When the author used mixed polarity enteroids, whose polarity had been partially reversed and contained both basal-out and apical-out surfaces, both pathogens preferentially invaded the apical side [39]. Apical-out human enteroids seem to be relevant and accessible models because they highlight the importance of cell polarity to visualize the mechanism of pathogen exit from the epithelium to promote shedding and dissemination. This is particularly true for pathogens that use basolateral receptors for invasion, such as *L. monocytogenes* or *S. flexneri*.

Organoids can be used to model the complex multicellular environment of the intestine. Experimental workflows now finely sum up the interactions of pathogens with highly specialized epithelia cells (i.e., mucus-producing cells, Paneth cells, and microfold (M) cells). This could overcome the limitations of the in vitro cell lines that commonly represent enterocytes [54]. 

The thick mucus layer is a key component of the physical barrier that protects the gut epithelium from the potential pathogens present in the luminal environment [63]. Transcript-based comparisons using organoids have showed changes in the expression signature of mucin *Muc2*, the major structural component of the intestinal mucus. A study based on fully differentiated enteroids infected with *S. flexneri* indicated the transcriptional upregulation of *Muc2* after apical or basolateral bacterial infection [64]. Similar *Muc2* transcript profiles were observed using the goblet-like cells HT29-MTX infected with *S. flexneri* [64]. While non-motile bacteria, such as *Shigella*, increased the level of *Muc2*, EHEC exposure to human colonoids reduce the thickness of the *Muc2*-positive mucus layer in less than 6 h [51].

The follicle-associated epithelium (FAE) is characterized by the presence of M cells, which constitute a niche for bacteria with an intracellular lifestyle because they naturally internalize foreign particles. M cells are exploited by many different pathogens, including *S. flexneri* [65], *L. monocytogenes* [66], and *S. enterica* Typhimurium [67], as a passage through the intestinal barrier to deeper host tissues [68]. *S. enterica* Typhimurium-infected enteroids derived from human small intestinal crypts confirmed that bacteria could rapidly trigger a transition from FAE enterocytes into M cells via an epithelial-mesenchymal transition (EMT) [69]. Similar findings were reported using cocultures of Caco-2 and Raji-B cells [70]. Stimulation with receptor activator of NF-κB Ligand (RANKL) and tumor necrosis factor alpha (TNF-α) was used to induce M cell differentiation in enteroids [71]. The resulting 3D intestinal in vitro device was used to study *S. flexneri* transcytosis via M cells [64]. The authors confirmed the presence of M cells using glycoprotein 2 immunostaining. *S. flexneri* invaded M cell-containing enteroids more often than it invaded non-stimulated enteroids [64]. 

FBDs are usually self-limiting and of short duration. Some FBD cases, however, can lead to long-lasting disability. A range of human tissues are currently expandable as organoids, but only a few applications are currently used to explore the interactions of FBPs with tissues or cells once the pathogen has colonized the deeper tissues. Organoids have been used to understand the molecular mechanisms behind the epidemiological association between chronic infection with *Salmonella enterica* and gallbladder carcinoma (GBC) in humans. Scanu et al. developed a murine gallbladder organoid (GBO) genetically predisposed to resemble the analogous *TP53* inactivation in GBC patients. Infected murine cells formed organoids in growth factor-free medium. In addition, they presented polarity loss and large irregular nuclei. These observations indicate a cell transformation driven by *Salmonella* infection [72]. More recent evidence reveals that the human restricted pathogenic serovar Paratyphi A induced DNA damage in human GBO [7]. A detailed analysis of longer-term infected organoids reveals that bacteria could drive the termination of cell replication via the downregulation of the transcriptional programs related to each cell cycle phase (G1/S, S, G2, and G2/M) [7]. Therefore, these studies showed not only a clear *Salmonella* tropism of gallbladder tissue, but also the underlying pathways of the connection between *S. enterica* and cancer.

## 4. Organoids for Investigating the Host Immune Response Following Foodborne Infection

Studying the interplay between FBPs and the distinct cellular populations in disease ecosystems also requires a large picture of the coordinated factors involved in the host defense mechanisms. Given the fact that the signature of organoids resembles the genetic signature of native intestinal epithelium cells and allows genome editing, organoids have also been used to study host signaling for maintaining a fine balance in the gut environment.

Studies have revealed the global transcriptional changes occurring within organoids during tissue inflammation and host defense. Forbester et al. identified a large spectrum of transcriptional changes by evaluating host–pathogen interactions with *S. enterica* Typhimurium [73]. Six of the most highly upregulated genes in the infected organoids consisted of genes related to the interleukins (ILs) that are essential messengers between immune cells and nonhematopoietic cells [73]. Karve et al. found no significant differences in the gene expression of proteins that are involved in gastrointestinal guarding between commensal *E.coli* and STEC strains. However, inflammatory mediators IL-8 and IL-18 were significantly upregulated upon STEC infection [52]. Organoids have also provided significant clues about host defense against *S*. *flexneri* infection. Elements of the NF-κB-mediated inflammation, including IL-8, TNF-α and TNFAIP3, were enriched in colonoid monolayers infected by *S. flexneri* [57]. Ranganathan et al. evaluated in more detail the effect of *S. flexneri* infection on IL-8 expression [64]. Enteroid and colonoid monolayers infected with *S. flexneri* secreted IL-8 in a time- and compartment-dependent manner. At the same time, the level of apical IL-8 was significantly higher than the level of basolateral IL-8 at the early phase of *S. flexneri* infection. At 26.5 h post infection, the level of basolateral IL-8 was higher than the level of apical IL-8 in the infected enteroids derived from either segment [64]. 

Although inflammasomes play diverse roles in innate immunity, their function in the central line of human defense against enteric pathogens has not been dealt with in depth. The big cytoplasmic multiprotein complexes can be activated by bacterial stimuli that unlock the canonical and non-canonical pathways, resulting in the secretion of IL-1β and IL-18 [74]. Moreover, the downstream effectors of inflammasomes are involved in activating signals of pyroptosis, a programmed form of cell death that occurs via IEC shedding [60]. Researchers have attempted to determine the role of each caspase in the defense against *Salmonella* infection. Murine enteroid infection models have showed a specific contribution of caspase-1 (*Casp1*) and caspase-11 (*Casp11*) (the equivalents of caspase-4 and caspase-5 in humans), which induced cellular responses and effector mechanisms. *Casp11*−/− and wild type (WT) enteroid-derived monolayers were much less passive upon *Salmonella* infection compared to *Casp1/11*−/− and *Casp11*−/− enteroid monolayers. This infection profile demonstrates that Casp-1 is sufficient to restrict bacterial invasion. Additional findings suggested that the proinflammatory response could upregulate *Casp-11* expression later in the course of infection, and that caspases acted together against pathogen attack [75]. In a similar fashion, Holly et al. compared the caspase-mediated activities of enteroids from human intestinal epithelium and mouse intestinal epithelium in response to infectious stimulation [50]. The human and murine enteroids responded to the microbe in a specie-dependent manner [50]. Whereas *Casp4*-deficient human enteroids completely stopped IL-18 secretion, the murine equivalent of *Casp4* (*Casp-11*) was found to be important but not essential. Similarly, the contribution of canonical and non-canonical pathways to decreasing the intracellular burden of *S. enterica* Typhimurium was species dependent. While non-canonical pathways play a key role in primary human cells, canonical pathways play a key role in primary mouse cells [50].

Forbester et al. generated organoids from healthy individuals and from a patient harboring a mutation in the IL10RB gene that inactivates the IL-22 receptor [35]. The IL-22 receptor expressed on the basal surface, and the subsequent IL-22 response occurred in organoids derived from healthy cells. In contrast, the IL10RB-defective organoids exhibited a loss of the IL-22 defense function. This highlights the relevance of this method for facilitating studies on phenotypic–genotypic associations. Further results demonstrated the infection-limiting mechanisms and a protective role of IL-22 via phagolysosomal fusion [35]. 

Beyond the understanding reached with organoids, integrating other cell types critical for intestinal homeostasis appears to be indispensable to mimicking the cellular microenvironment. A reliable model of the crosstalk between immune cells and IEC was created by Noel et al. [42]. The macrophages introduced in the basolateral compartment of a mixed enteroid monolayer system developed the ability to cross the intestinal epithelium without harming the medium upon which they were engrafted [76]. Noel et al. observed the reactions of the human macrophage–enteroid coculture in response to a bacterial stimulus on the apical surface [42]. The number of CFUs in the upward phase of enteroxigenic *E. coli* (ETEC) in the pathogen hybrid coculture was significantly lower than in the macrophage-free enteroids as early as 30 min post-infection [42]. Given that fact, this experiment reflects the successful sensing and bactericidal activity of macrophages. The coordinated work of the intestinal barrier and mucosal immunology to prevent infection of the human gut was also accompanied by lower pro-inflammatory cytokine secretion, including IL-8, IL-6, and IFN-γ [42]. On a wider scale, future studies should deal with mechanistic observations of macrophage transepithelial projections and their contact with enteric pathogens [42]. In the same vein, polymorphonuclear leukocytes (PMN) were added to wells containing organoids, mirroring neutrophil recruitment during EHEC infection on the luminal surface. Images of the control and transcriptional profiles identified PMN cells in the external edge of organoids and the upper production of IL-8, respectively [52], which is known to favor PMN cell attraction. IL-8 is also a key factor in neutrophil recruitment in animal enteric infection model [77]. These results represent an excellent initial step toward increasing the complexity of organoids by including stromal elements.

Incorporating genetic engineering into organoid technology could provide further knowledge on the host factors that influence the functions of the intestinal barrier and intestinal defense mechanisms, and, finally, lead to the development of enteric diseases. For instance, mutated organoids that reflect specific tissue phenotypes have facilitated in-depth experimentation to further analyze infection mechanisms. In 2015, Wilson et al. compared the antimicrobial activity of α-defensins in the epithelial defense against *S. enterica* Typhimurium replication using organoids derived from wild-type and mutated mouse cells for α-defensin production [78]. Comparative assays demonstrated that intra-luminal *S. enterica* Typhimurium growth was significantly higher in the deficient genotype model. The intestinal ex vivo system may compensate for the anti-bacterial activity through the expression of human defensin HD5 [78]. 

In addition to cell host responses to infection, organoid tools can also address infection mechanisms on the bacterial side.

## 5. Organoids for Studying the Virulence Mechanisms of FBPs

Microorganisms possess a number of interlinked virulence traits that constantly move toward the establishment of infection and which trigger disease and their persistence in the host. The study of pathogen effectors may lead to the development of new rapid diagnostics tools or detection methods, therapeutic drugs, and vaccines to better control foodborne pathogens. Organoids are paving the way for additional and promising investigations of molecular aspects of FBP virulence.

The engineering of genes that encode virulence effectors and host adaptation may well be the keystone to fully understanding the causality between a gene defect and infection developed in organoids.

Interestingly, using enteroids, Geiser et al. attempted to describe the *S. enterica* Typhimurium cycle of infection, and uncovered novelties about the role of known virulence factors [79]. *S. enterica* pathogenesis involves the type three secretion system 1 (TTSS-1), which mediates the translocation of effector proteins into host cells to promote bacterial invasion. According to the authors, TTSS-1 activity and some TTSS-1 effectors (SipA, SopB, SopE, and SopE2) seem to promote *S. enterica* Typhimurium colonization in human enteroids by enabling the bacterial invasion of intestinal epithelial cells. However, flagellar motility does not seem to be required for the efficient bacterial colonization of enteroids; *Salmonella* seems to reach the epithelial surface and invade the intestinal epithelial cells through gravitational sedimentation within enteroids [79].

Intestinal organoids could also be an important tool to shed more light on microbial inter-strain—and even inter-serovar—variation in pathogenicity. For example, infected human ileum-derived organoids were used to evaluate the serovar specificity of disease phenotypes to help analyze the role of the YrbE phospholipid transporter in *S. enterica* Typhi and Typhimurium. Verma et al. established that deletion of the *yrbE* gene induced several changes in *S. enterica* Typhy bacteria, such as the over-expression of flagellin, resulting in uncontrolled motility, elevated IL-8 secretion, and deficient adherence to the organoid of the mutant strain. In contrast, *S. enterica* Typhimurium pathovar did not seem to be affected by the disruption of *yrbE*. These results suggest that YrbE might be involved differently in the pathogenic mechanisms of *S. enterica* serovars, especially in the early steps of infection [80].

A neglected field of study using the overly simplistic 2D models has been the molecular routes likely to be involved in the watery diarrhea that is triggered by the majority of FBPs that colonize the human intestinal epithelium. Based on the advances of culture systems, Tse et al. recreated a colonic environment to evidence the potential enterotoxic effect of extracellular serin protease P (EspP) excreted by EHEC, which displays electronic transport and therefore leads to diarrhea [81]. Measuring changes in active ion movements in human colonoid monolayers, the authors indeed detected a significantly increased transport of colonic electrolytes related to EspP luminal concentrations. Thus, additionally to its protease activity, EspP may be a factor involved in EHEC diarrheic episodes [81]. Broader research should investigate the role of serine protease activity from other enteric infectious agents in organoid-pathogenic phenotypes [82].

A study using organoids derived from intestinal tissue taken from human biopsies revealed novel insights into *S. enterica* Typhi small intestinal mucosa infection. A transmission electron microscopy (TEM) analysis indicated a cytoskeletal change, with microvilli destruction leaving a more accessible surface for pathogen entry and vesicle-contained intracellular bacteria. Secondly, while *S. enterica* Typhimurium invasion predominantly occurred through M cell-facilitated phagocytosis, *S. enterica* Typhi infection mostly progressed via the enterocytes [83].

The characterization of the host cell invasion mechanisms and of the effect of pathogens on intestinal stem cells was studied in *Listeria* organoid models. Co et al. confirmed previous findings that *L. monocytogenes* preferentially binds to basolateral receptors to invade intestinal cells [39]. This bacterium targets sites of cell extrusion, where basolateral proteins are apically exposed, and enters the apical epithelium in human enteroids [39]. Five hours post-infection, *L. monocytogenes* translocated in greater numbers across the distal small intestine epithelial monolayers derived from organoids than they did across the proximal monolayers [84]. In addition, invasion by *L. monocytogenes* altered the morphology of the intestinal organoids, especially the intestinal stem cells, and reduced the budding rate [85]. *L. monocytogenes* modulated organoid proliferation by regulating stem cell niches, which disrupted normal intestinal turnover [85]. In addition, this pathogen affected the expression of *Hes1*, *Math1*, and *Sox9*, and this interfered with the differentiation of intestinal stem cells [85]. Besides investigating the molecular mechanisms associated with the enteritis caused by foodborne pathogens, some works have used organoid/enteroid models to explore the other pathologies induced by these pathogens. For example, *Campylobacter jejuni* is known to be the major cause of bacterial enteritis worldwide. Moreover, *Campylobacter* spp. have been observed in patients with colorectal cancer (CRC), and has been associated with the development of inflammatory bowel disease, a known risk factor of CRC [86,87,88,89]. He et al. demonstrated that the human clinical isolate *C. jejuni* 81–176 promotes colorectal tumorigenesis through the action of cytolethal distending toxin (CDT) [90]. The key role of CDT in this process was showed using various models, such as mice (germ free *Apc^Min/+^*), a non-transformed rat small intestine epithelial cell line (IEC-6), a human colon cancer cell line (HT-29), and cultured enteroids [90]. Cultured enteroids were used to evaluate the effect of *cdtB* on DNA damage in primary intestinal cells. Exposure of enteroids to *C. jejuni* lysates enhanced γH2AX induction (a surrogate marker of DNA damage) compared with the control, while this response was attenuated in enteroids exposed to *C. jejuni* with an inactivated *cdtB* gene [90]. These findings demonstrate that *cdtB* plays an important role in *C. jejuni*-induced DNA damage and cell cycle arrest in vitro.

## 6. Using Organoids to Investigate the Anti-FBP Activities of Probiotic (-like) Bacterial Strains

Organoids are receiving much attention due to their high resemblance to the physiology of the gastrointestinal environment. They have not showed their full potential yet, and there are still shortcomings when modeling complex environments, such as the intestinal microbiota. However, they provide the initial steps toward a more refined understanding of potential microbe-based therapies, such as probiotics. This fact is consistent with the widespread interest in the development of a robust line of new drugs and innovative pathways to bring solutions to patients suffering from either drug-resistant bacterial infections or—even more critically—infectious diseases with only supportive treatment (i.e., EHEC infections). 

The commensal strain *E. coli* Nissle has been used as a probiotic for more than a century, and, more recently, to treat intestinal disorders. However, this strain is highly related to a pathogenic *E. coli* strain isolated from a patient with pyelonephritis [91]. Pradhan and Weiss have used human intestinal organoids to assess the safety and protective effects of the probiotic strain against *E. coli* pathogenic strains [92]. In single-strain infection studies, Nissle did not cause damage to organoids. However, in co-infection experiments, Nissle protected organoids from the EHEC-mediated loss of the epithelial barrier function and EHEC-induced apoptosis [92]. The results also suggest that Nissle can be vulnerable to phages and that lysogens can produce the Shiga toxin, which would limit the usefulness of the probiotic as a therapeutic alternative [92]. 

Introducing potential probiotic microbes into organoids has recently emerged from disease mimicking based on the crosstalk between microbial components and their microenvironment. In 2020, Lu et al. investigated the use of *Lactobacillus acidophilus*, a recognized probiotic microorganism, to drive protective mechanisms on the gut barrier exposed to *Salmonella* [93]. Pre-treatment with the *L. acidophilus* caused more active mucus secretion, resulting probably from the general IEC response to contact with microorganisms [93]. Furthermore, *L. acidophilus* modulated toll-like receptors (TLRs), which are involved in the hyperplasia and inflammation caused by *Salmonella* infection [93]. In the same way, the ability of five lactic acid bacteria strains to modulate the vitamin D receptor (VDR) pathways and *S. enterica* enteritidis-induced inflammation and infection was evaluated using murine organoids [94]. Some of these strains protected organoids from *Salmonella* inflammation by increasing VDR expression [94]. In addition, VDR deletion in organoids resulted in more severe inflammation and bacterial invasion upon *Salmonella* infection [94].

The well-orchestrated communication between epithelial and non-epithelial cells is essential to decipher the arsenal of infection-related responses set up by the host. In the particular case of foodborne infections, gut immunology, for instance, plays a crucial role in maintaining the host–microbiota interactions, and it is interesting to elucidate the crosstalk between the intestinal epithelium and immune cells.

## 7. Current Challenges and Future Prospects

*In-depth investigation of pathogenic mechanisms.* The evolution of cell models towards the design of structures that approximate the real microenvironment to which pathogens are exposed in the gut is still of interest in order to improve the understanding of host–pathogen interaction. For example, *Campylobacter jejuni* is unanimously recognized as the leading cause of bacterial enteritis in the world. Paradoxically, however, despite numerous studies on animal and “traditional” cell models, its pathogenic mechanism has still not been fully described. It seems that the models used so far do not sufficiently reproduce the relationship between the bacteria and intestinal cells. The mechanism of *C. jejuni* translocation is especially controversial and not well understood. Consequently, enteroids are therefore likely to investigate more deeply the transmigration of *C. jejuni* across the intestinal epithelium and to provide new information on intestinal campylobacteriosis. In addition, using intestinal organoids from livestock animals can help to investigate the host specificity of zoonotic bacteria in a one health context [95,96]. In addition, new approaches for improving the accessibility of the pathogen to the apical surface of organoids have been investigated. A robotically articulated microinjection platform showed enhanced performance by transporting a bacterial suspension at a rate of approximately 90 organoids per hour. Nevertheless, the efficiency of the device varied considerably due to great organoid heterogeneity in terms of size, shapes, luminal volumes, and monolayer width [97].

*Increasing model complexity to assess interactions of FDP with other organs and the environment.* Intestinal organoids are mainly exploited as single-organ systems representing the gut epithelium, lacking for mesenchymal or immune cell populations naturally present in the gut mucosa. In order to better model human disease and to evaluate the role of the mucosal compartment and epithelial–immune cell communication occurring in FPD, cocultures of epithelial organoids with other organ-specific elements are of interest, such as with macrophages and T cells. The cellular diversity gain from organoids can also be exploited by interconnecting multiple organ systems in fluidic systems under dynamic conditions. Organ-on-chip devices that use organoids derived from stem cells can model multi-organ complexity, such as the gut–brain axis or the interaction between the gut and kidney, allowing for the study of infection progression from primary to secondary infection sites. In addition, this “organoids-on-chip” technology can reproduce the mechanical forces to which the enteric pathogens can be exposed in the intestinal environment, such as flow and peristalsis. These mechanical constraints seem essential for infectivity. 

*Towards personalized medicine in foodborne infectious diseases?* One of the most pressing clinical challenges is developing precision medicine in FBP infection. Biobanks can be built using enteroids from different normal or genetically and clinically diverse individuals to facilitate fundamental research, but also to study the effect of pharmacological compounds in a heterogeneous population. Existing human intestinal organoid biobanks derived from healthy and diseased tissues have been established, especially from cancers, but also other diseases, such as inflammatory bowel disease and cystic fibrosis [41,45,98]. Co-clinical trials have already been performed to confirm the usefulness of organoids in drug screening by comparing them with other models (e.g., animal models) and with patients’ responses, showing in vitro to in vivo correlations [99,100,101]. Most applications of organoids for precision medicine are currently related to the screening of anticancer therapeutics. These biobanks can be used for high-throughput screening assays to assess the efficacy and toxicity of drugs in a personalized fashion. The genetic engineering of organoids or patient-derived organoids harboring mutations related to pathogenic bacterial infections may disclose the potential associations between genetic signatures and susceptibility to infectious diseases, and can be used to predict responses to drugs. However, the use of human organoids to fully understand infectious diseases requires the development of technologies that are sufficiently simple for routine use in infectious disease laboratories and adequately robust for use in preclinical studies. The addition of a functional immune system, a complete microbial influence, and the generation of M cells remain to be optimized. Moreover, the generation of standardized protocols and mainstream organoid media will make the model more accessible for laboratories and clinics willing to adopt the model and to provide more accurate data. 

## 8. Conclusions

Over the past decade, organoids have appeared that could act as a human model for studying the virulence of enteric bacterial pathogens. To move closer to in vivo pathophysiological mechanisms, the next stage of disease modelling using organoids will require more complex and robust strategies. Recent evidence has revealed that introducing non-epithelial cells, e.g., microbiota and immune cells [42,97] (Figure 1g,h), and improving pathogen attachment through more refined techniques, such as microinjection techniques, apical phase reversion, or using primary epithelial cell monolayers, may considerably empower the study of interactions of the intestinal ecosystem–pathogen interface using organoids. As the complexity of these model systems increases with cocultures and organ-on-chip systems, new opportunities and challenges arise, and the host–pathogen interaction landscape will benefit from them.

## Figures and Tables

**Table 1:** Comparison of 2D versus 3D cell cultures (as reviewed in [28,29,30]). The phrase 2D cell culture refers to monolayer epithelial cells (not derived from organoid/enteroid models), whereas 3D cell culture refers to organoid and enteroid models.

Comparison	2D Monolayer Cell Culture	3D Cell Culture
Cell differentiation into enterocyte or goblet cell	✓	✓
Cell differentiation into Paneth cell and enteroendocrine lineages	-	✓
Easily accessible to the apical side of cells	✓	-
Include immune, nerve, or vascular cells	-	-
Cell polarisation	✓	✓
Formation of cell–cell tight junctions	✓	✓
Development of villus-like and crypt-like structures—three-dimensional architecture	-	✓
Expanded indefinitely	✓ (if derived from tumour cells)	✓
Cryopreservation for long-term storage	✓ (if derived from tumour cells)	✓
Reproducibility	+++	+
Cost	+	+++

Legend: (✓), presence. (-), absence. (+), low. (+++), high.

**Table 2:** Main advantages and disadvantages/limitations of 3D cell cultures (as reviewed in [45,46,47,48,49]).

Advantages	Disadvantages
Better mimic endogenous tissues, including organization and spontaneous differentiation of multiple cell types into physiologically relevant 3-D structures, expression and localization of tight junctions, mucus production, polarity, gene expression, cell viability and proliferation, cytokine production	Heterogeneity in size, shape, and viability of organoids within a culture and across different samples, owing to the diversity of individuals and protocols. Protocols for organoid establishment and quality control are not globally standardized.
Contain highly polarized cells that differentiate into the cell lineages of the tissue of origin, i.e., intestinal organoids contain fully mature goblet cells, enterocytes, Paneth cells, and enteroendocrine cells.	Lack of neural innervation, immune cells, vasculature, and amicrobiome → coculture systems with other cell types are not firmly established. Lack of mechanical stress (peristalsis) and luminal and basolateral flow → towards organoid on chip.
Personalization: induced pluripotent stem cells and organoids can be obtained from individuals	Infection experiments: closed system that represents a nonphysiological route for pathogens that infect via the apical/luminal side, i.e., the luminal side is inaccessible without microinjection or disruption of organoid polarization. Microinjection remains a technical challenge.
Genetic engineering: most modern genetic engineering tools can be applied to induced pluripotent stem cells or directly to organoid systems	Relatively costly: organoids cost less than animal models, but they are relatively expensive compared to traditional cell lines (mainly due to medium composition with growth factors and volume required for culturing large numbers of cells).

In the following sections, the main studies related to the use of organoids to decipher the virulence mechanisms of FDPs and the responses of the host cells are discussed.

## Data Availability

Not applicable.
